# Moracin M inhibits EV71 infection primarily by blocking viral attachment to host cells

**DOI:** 10.1128/aac.01932-25

**Published:** 2026-06-04

**Authors:** Yinying Zhu, Qian Peng, Lijuan Hu, Weiling Li, Yingying Shi, Xiansheng Ye, Xiji Shu, Yuchen Liu, Wei Liu, Pin Wan, Binlian Sun

**Affiliations:** 1 Hubei Key Laboratory of Cognitive and Affective Disorders, Institute of Biomedical Sciences, School of Medicine, Jianghan University 470004, Wuhan, China; 2 Hubei Provincial Demonstration Center for Experimental Medicine Education, School of Medicine, Jianghan University 470004, Wuhan, China; 3 Department of Immunology, School of Medicine, Jianghan University 470004, Wuhan, China; Chinese Academy of Medical Sciences & Peking Union Medical College, Beijing, China

**Keywords:** HFMD, Moracin M, EV71, CA16, viral attachment

## Abstract

The global outbreak of hand, foot, and mouth disease (HFMD), mainly caused by enterovirus A71 (EV71) and coxsackievirus A16 (CA16) of the genus enterovirus, poses a serious threat to the health of young children, and severe neurological manifestations and associated symptoms are primarily attributed to EV71 infection. There is currently no specific antiviral drug available for HFMD. In this study, we identified Moracin M (MM), a flavonoid compound derived from *Smilax china*, as a potent inhibitor of EV71 and CA16 infection. Mechanistic investigation revealed that MM exerts its antiviral effects primarily by inhibiting viral attachment to host cells by targeting the virus. Furthermore, MM showed excellent safety and significant anti-EV71 activity in neonatal mice, and it also effectively suppressed the inflammatory response and alleviated muscle, lung, and brain tissue damage induced by EV71 infection. In conclusion, our study identified and demonstrated MM as a promising inhibitor of EV71 and CA16, providing important insights for the further development of MM as a potential therapeutic agent for HFMD caused by these viruses.

## INTRODUCTION

Cyclical epidemics of hand, foot, and mouth disease (HFMD) caused by human enterovirus infections predominantly affect the health of children under 5 years of age (1). It has been reported that enterovirus A71 (EV71) and coxsackievirus A16 (CA16), the members of genus enterovirus, are the primary pathogens responsible for HFMD. HFMD outbreaks have been frequently reported worldwide, and it is estimated that approximately one million infants and young children suffer from HFMD annually in China by 2023 (2). These enteroviruses initially infect the pharynx and intestine and then disseminate throughout the body via the bloodstream. The primary clinical manifestations of HFMD are rashes and blisters on the hands, feet, and oral mucosa (3); some patients rapidly develop severe neurological manifestations, such as brainstem encephalitis, pulmonary edema, and acute flaccid paralysis (3–5). Currently, the common antiviral agents ribavirin, oseltamivir, and glucocorticoids are employed in the treatment of HFMD (6), but still no specific antiviral agents are available for the treatment of enterovirus-associated diseases.

EV71 and CA16 particles exhibit an icosahedral structure with a diameter of approximately 30 nm (7), and their genome, a single-stranded positive-sense RNA molecule approximately 7.4 kb in length, is enclosed within a protein capsid. The viral capsid is composed of four structural proteins, among which VP1, VP2, and VP3 are exposed on the outer surface, while VP4 is located internally within the capsid (8). VP1 plays a critical role in viral recognition of cell surface receptors and directly determines the antigenicity of EV71 and CA16 (8, 9) and is usually used as a marker for detecting the level of viral infection. The early stages of the viral life cycle include virus attachment and entry into target cells, which involve the initial interaction between the virus and the host cell (10, 11). Following entry, viral genome replication and protein synthesis facilitate virus proliferation and dissemination, which are susceptible to interference by host factors (12).

Currently, drugs derived from natural products are widely used in clinical practice (13–15). For example, artemisinin, extracted from Artemisia annua, is primarily used for the treatment of malaria (16); paclitaxel, extracted from the bark of Taxus chinensis, is mainly used to treat ovarian and breast cancer (17); glycyrrhizic acid, extracted from licorice, is primarily used for the treatment of hepatitis and skin allergies (18). *Smilax china* is widely used in Chinese medicinal formulas due to its various pharmacological effects, including anti-inflammatory, anti-bacterial, anti-tumor, and immunosuppressive activities (19). Moracin M (MM), which was isolated from *Smilax china* by our group, exists in many plants as a flavonoid compound, which possesses multiple biological activities. Studies have demonstrated that MM can suppress inflammatory responses and alleviate oxidative stress (20, 21); MM and its derivatives, such as Moracin P and Moracin O, exhibit inhibitory effects on the NS3 protease of HCV (22). The anti-enterovirus activity of MM has not yet been reported.

In this study, we identified and demonstrated that MM significantly inhibits the infection of EV71 and CA16; further investigation revealed that MM primarily inhibits the attachment of these viruses to target cells by directly acting on the viral particles rather than on the cells. Furthermore, MM exhibits good safety and a significant anti-EV71 effect in newborn mice. These findings suggested that MM has the potential to be developed as a therapeutic or prophylactic agent for HFMD caused by EV71 and CA16 infection.

## MATERIALS AND METHODS

### Cells, viruses, and mice

Human embryonal rhabdomyosarcoma (RD) cells, African green monkey kidney epithelial (Vero) cells, human neuroblastoma (SH-SY5Y) cells, and human glioblastoma (U87) cells were maintained in our laboratory and were cultured in complete Dulbecco’s modified Eagle medium (DMEM, Gibco, USA) supplemented with 10% fetal bovine serum (FBS, Gibco, USA) under a humidified atmosphere at 37°C and 5% CO₂.

EV71 containing green fluorescent gene (EV71-GFP) was a kind gift from professor Bo Zhang (Wuhan Institute of Virology, Chinese Academy of Sciences); the wild-type EV71 strain (LYG03, GenBank accession no. PQ015377) and a CA16 strain (CA16-#3, GenBank accession no. PX095330) maintained in our laboratory were used to evaluate the antiviral activity of MM. The virus was propagated in RD cells as follows; once obvious cytopathic effects (CPE) appear in the virus infected cells, the supernatant containing the virus is harvested, and cell debrises were then removed by centrifugation, followed by filtration through a 0.45 μm filter (HABG02500, Millipore). Viral titers were determined by the 50% tissue culture infectious dose (TCID₅₀) assay. In this study, the cells were primarily infected at a multiplicity of infection (MOI) of 1 or 0.5 or 5, defined as the ratio of infectious virus particles to target cells at the onset of infection. All virus amplification and infection experiments were performed in a BSL-2 laboratory.

Specific pathogen-free (SPF) ICR pregnant mice were purchased from Vital River (Beijing, China) and maintained under standard conditions with a 12 h light-dark cycle and free access to food and water. Newborn suckling mice were used for experiments at postnatal day 3. All experimental procedures involving animals were conducted in accordance with approved protocols and guidelines established by the School of Medicine, Jianghan University (approval no. JHDXKJLL2025-128).

### Compounds and antibodies

We isolated 31 small-molecule compounds from the methanol extract of the medicinal plant *Smilax china* (23). The purity of all compounds exceeded 95% as determined by high-performance liquid chromatography (Agilent, CA); they were dissolved in dimethyl sulfoxide (DMSO) at a stock concentration of 50 mM and subsequently diluted with DMEM medium containing 10% FBS. Screening for EV71 inhibition with these compounds revealed that SC-11 exhibited significant antiviral activity, and its structure was identified as Moracin M (MM) by nuclear magnetic resonance analysis. MM used in subsequent experiments was all purchased (HY-122942, MCE) and dissolved in DMSO at a stock concentration of 200 mM. Ribavirin, a well-known nucleotide analog with broad-spectrum antiviral activity, was used as the positive control (HY-B0434, MCE) and dissolved in DMSO at a stock concentration of 100 mM. For experimental use, compounds were diluted to working concentrations in cell culture medium, with the final DMSO concentration maintained below 0.1% (vol/vol) in all assays.

The rabbit anti-VP1 polyclonal antibody was prepared in our laboratory and can recognize the VP1 protein of both EV71 and CA16, the rabbit anti-3D (A8608, ABclonal) can recognize the 3D protein of EV71, and the mouse anti-GAPDH antibody used as internal control (60004-1-Ig, Proteintech). Goat anti-mouse IgG-HRP (BA1055, Boster) and Goat anti-rabbit IgG-HRP (BA1051, Boster) were used as second antibody diluted at 1:3000.

### Inhibitor screening with EV71-GFP

For EV71 inhibitor screening, RD cells were seeded in 12-well plates at a density of 2 × 10⁶ cells per well and cultured overnight, followed by infection with EV71-GFP at an MOI of 1 for 2 h. After washing with PBS, cells were further cultured in fresh medium containing the test compounds at 50 μM or ribavirin at 80 μM for 24 h. Following a second PBS washing, cells were harvested and fixed with 4% paraformaldehyde (BL539A, Biosharp) for 30 min. The proportion of GFP-positive cells (FITC signal) was analyzed by flow cytometry using a C6 Plus instrument (BD, USA). The inhibition rate (%) was calculated using the following formula: [1 − FITC (treatment)/FITC (vehicle control)] × 100%.

### Determination of viral TCID_50_

The TCID₅₀ of the virus in the supernatant of EV71 infected cells was determined by the Reed-Muench method (24). Briefly, Vero cells were seeded in 96-well plates and cultured for 12 h. The EV71-containing supernatant was serially diluted 10-fold with DMEM without FBS, and 10 μL of each dilution was added to cells in sequence. Cytopathic effects were monitored until the experimental endpoint, defined at which cells became rounded and began to undergo massive detachment. TCID₅₀ was then calculated according to the Reed-Muench method (25).

### Cell toxicity assay

RD and Vero cells were seeded in 96-well plates at a density of 1 × 10⁴ cells per well and treated the next day with different concentrations of MM (ranging from 0 to 200 μM) for 24 h. Subsequently, 10 μL of CCK-8 reagent (C0038, Beyotime) was added to each well and was incubated for 1 h at 37°C; then, the absorbance of medium was measured at 450 nm using a multimode microplate reader (Multiskan FC, Thermo Fisher). The cell viability was calculated as the percentage of absorbance of MM treated cells relative to vehicle control.

### Antiviral activity assay

RD and Vero cells were seeded in 12-well plates at a density of 2 × 10⁶ cells per well and cultured overnight. After infection with EV71-GFP (MOI = 1), EV71-LYG03 (MOI = 0.5), or CA16-#3 (MOI = 1) for 2 h, the cells were washed and further cultured in medium containing serially diluted MM (ranging from 0 to 50 μM) for 22 h. GFP expression was observed using a fluorescence microscope, and the proportions of GFP-positive cells were quantified by flow cytometry. The antiviral activity of the compounds was defined as the half-maximal inhibitory concentration (IC₅₀), which was calculated using GraphPad Prism software (version 10.0). For viral proteins and viral RNA measurement, cells under the same treatment conditions were lysed with RIPA buffer (P0013C, Beyotime) and TRIzol reagent (15596026, Invitrogen), respectively.

### Real-time quantitative PCR

Total RNA was isolated from samples using TRIzol reagent according to the manufacturer’s protocol. Two micrograms of RNA was used for cDNA synthesis with M-MLV Reverse Transcriptase (2641Q, Takara). Real-time quantitative PCR (RT-qPCR) was performed using TB Green Premix Ex Taq II FAST qPCR Mix (RR420Q, Takara) on a CFX 96 Real-Time PCR Detection System (Bio-Rad Laboratories, USA). The GAPDH gene was used as the internal reference control. The primer sequences used in this study are listed in Table 1.

**TABLE 1:** The primer sequences used for RT-qPCR analysis

Primer	Sequence
EV71-VP1-F	5′-GCAGCCCAA AAGAACTTCAC-3′
EV71-VP1-R	5′-ATTTCAGCAGCTTGGAGTGC-3′
Human GAPDH-F	5′-GCACCGTCACGGCTGAGAAC-3′
Human GAPDH-R	5′-TGGTGAAGACGCCAG TGGA-3′
Mouse GAPDH-F	5′-TTCACCACCATGGAGAAGGC-3′
Mouse GAPDH-R	5′-GGCATCGACTGTGGTCATGA-3′
Mouse IL-1β-F	5′-GCTGCTTCCAAACCTTTGAC-3′
Mouse IL-1β-R	5′-AGCTTCTCCACAGCCACAAT-3′
Mouse IL-6-F	5′-CCACTTCACAAGTCGGAGGC-3′
Mouse IL-6-R	5′-GGAGAGCATTGGAAATTGGGGT-3′
Mouse TNFα-F	5′-CCGATGGGTTGTACCTTGTC-3′
Mouse TNFα-R	5′-CCGATGGGTTGTACCTTGTC-3′
Mouse CXCL10-F	5′-ATGACGGGCCAGTGAGAATG-3′
Mouse CXCL10-R	5′-CGGATTCAGACATCTCTGCTCAT-3′

### Western blot assay

Western blot was performed following our published paper (26). Briefly, the cells were harvested and lysed using RIPA buffer (Beyotime, P1045) containing protease inhibitors. The protein concentration in the cell lysates was quantified using a BCA protein assay kit (Boster, AR1189), and 30 µg protein was separated by 10% SDS-PAGE and then was transferred onto PVDF membranes. The membranes were blocked with TBST containing 5% non-fat dried milk for 1 h at room temperature, followed by incubation with primary antibodies overnight at 4°C, after washing incubated with horseradish peroxidase (HRP)-conjugated secondary antibodies for 1 h at room temperature. Finally, protein bands were visualized using a ChemiDoc XRS Gel Imaging System (Bio-Rad Laboratories, USA) with enhanced chemiluminescence (ECL) reagents (Beyotime, P0018M).

### Viral attachment, entry, and replication assays

For the viral attachment assay, RD cells were pre-chilled at 4°C for 2 h and then incubated with the pretreated EV71-GFP or CA16-#3 (MOI = 5) with 50 μM MM at 4°C for 1 h. The cells were then washed three times with ice-cold PBS to remove the unattached virus, followed by RNA extraction and RT-qPCR to detect viral RNA. For the viral entry assay, RD cells were incubated with EV71-GFP or CA16-#3 (MOI = 5) at 4°C for 1 h to allow viral attachment. After three washes with ice-cold PBS to eliminate the unattached virus, cells were treated with 50 μM MM at 37°C for 1 h to permit internalization. Finally, the cells were washed with PBS and processed for viral RNA detection by RT-qPCR. For the viral replication assay, RD cells were infected with EV71-GFP (MOI = 1) at 37°C for 2 h. Following the removal of uninternalized virus by washing, cells were maintained in fresh culture medium supplemented with 50 μM MM and incubated at 37°C for 4h, and then the cells were collected and subjected to viral RNA detection by RT-qPCR.

### Evaluation of drug safety and antiviral activity *in vivo*

The acute toxicity study was performed on the basis of a previous report (27), and the dose of MM *in vivo* was used according to the previous report (20). Three-day-old ICR neonatal mice were randomly divided into three groups (10 mice per group): the control group (PBS), the high-dose group (MM, 20 mg/kg), and the triple-dose group (MM, 60 mg/kg). Mice were administered MM intraperitoneally at doses of 20 or 60 mg/kg/day for 7 consecutive days, the body weight was monitored daily, and the survival rate was calculated. On day 8, the mice were anesthetized with isoflurane and carried out euthanasia by dislocating the spine, and the liver, kidney, and spleen were immediately harvested, rinsed with cold saline, blotted dry with absorbent paper, and weighed. Organ coefficients, the ratio of the weight of a specific organ to the total body weight of the mouse, were calculated as a valuable indicator for detecting potential physiological abnormalities in visceral organs induced by acute drug exposure. The relative organ weight (ROW) for each animal was calculated as follows: ROW = [absolute organ weight (g)/body weight on the day of sacrifice (g)] × 100. Subsequently, the organs were fixed in 4% paraformaldehyde for histopathological analysis with Hematoxylin and Eosin (H&E) staining. Histopathological changes in both control and treatment groups were examined and documented under a light microscope.

The *in vivo* efficacy of MM was evaluated using ICR newborn mice. EV71 infection mice experiments were conducted in the ABSL-2 laboratory of Wuhan Institute of Virology, Chinese Academy of Sciences, and the established protocols were followed throughout the study in compliance with ABSL-2 biosafety and ethical guidelines. Three-day-old neonatal ICR mice were randomly assigned to three groups (6 mice per group): the non-infected group, the infected group, and the post-infection drug-treated group. Twelve mice were intraperitoneally injected with 10 μL of EV71-LYG03 (10⁷ TCID₅₀), while 6 mice were injected with 10 μL of DMEM as control. Subsequently, the infected mice were randomly divided into two groups. One group was intraperitoneally injected with MM (20 mg/kg) daily, while the other group, along with the uninfected mice, received PBS treatment for 5 consecutive days. Hind-limb muscle, lung, and brain tissues were collected from all mice at 6th day post-infection (dpi). Part of the tissues was fixed in 4% formaldehyde for H&E staining, and other part of the tissues was used for total RNA extraction, followed by the determination of viral RNA and the mRNA levels of inflammatory factors using RT-qPCR.

### Statistical analysis

Data were analyzed using GraphPad Prism 10 software (CA, USA). All data are presented as mean ± standard error of the mean (SEM) from three independent experiments. Statistical significance was assessed by one-way ANOVA followed by Tukey’s multiple comparisons test and unpaired *t*-test. A *P* value of less than 0.05 was considered statistically significant.

## RESULTS

### Compounds screening implied MM containing anti-EV71 capacity

**Fig 1:**
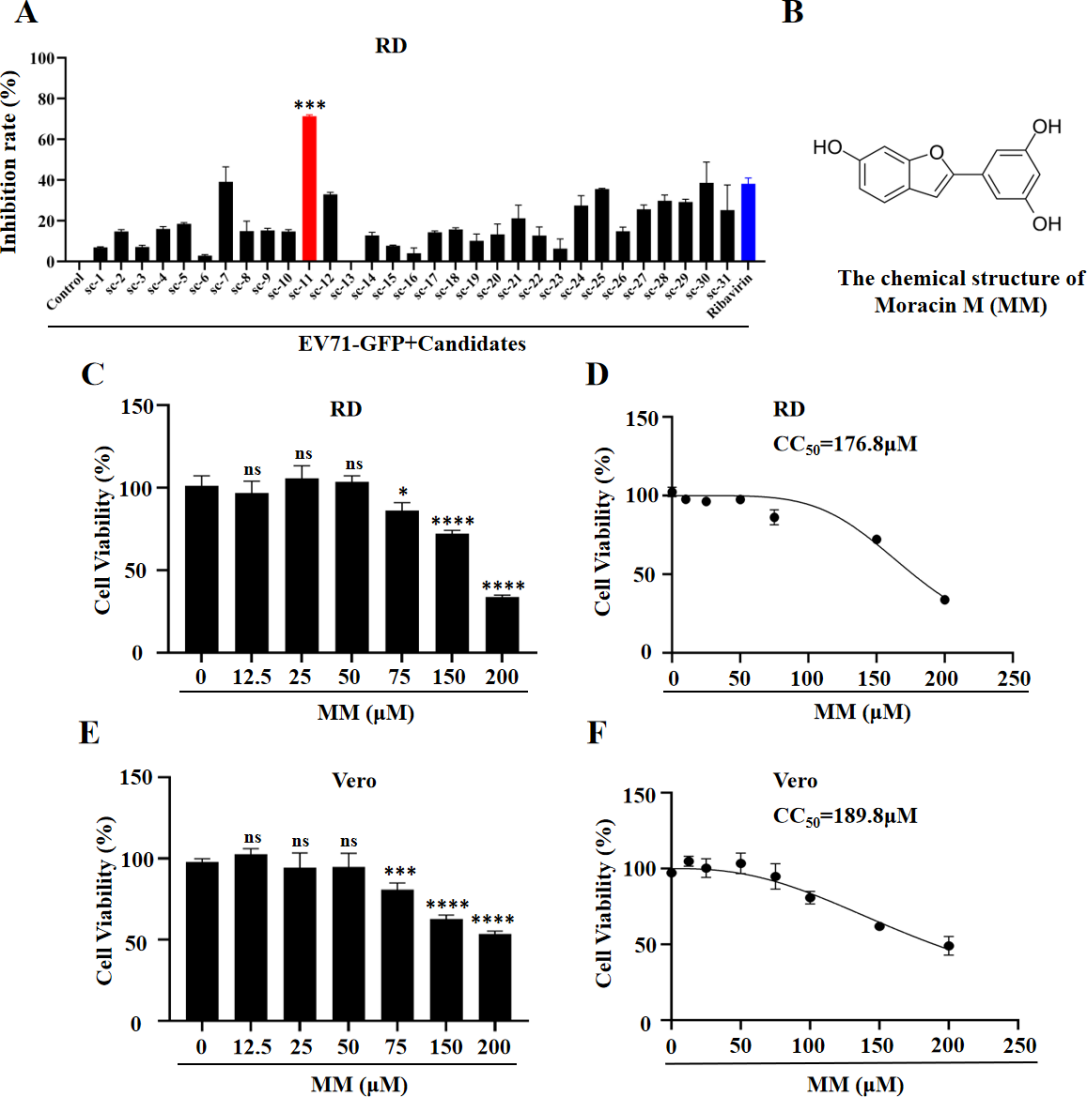
Screening of EV71 inhibitors using EV71-GFP. (**A**) RD cells were infected with EV71-GFP for 2 h in 12-well plates. Subsequently, the supernatant was replaced with fresh medium containing 31 candidate compounds, respectively (50 μM each). Ribavirin (80 μM) was used as a positive control, and 0.1% DMSO served as the vehicle control. The percentage of GFP-positive cells after 24 h of treatment was quantified by flow cytometry, and the relative inhibition rate of compound MM was calculated by comparing with the vehicle control, and the inhibition rate of SC-11 was compared with that of ribavirin. (**B**) Chemical structure of compound MM. RD cells (**C**) and Vero cells (**D**) pre-seeded in 96-well plates were treated with increasing concentrations of MM (0–200 μM) for 24 h. Cell viability was assessed using the CCK-8 assay, and the CC_50_ values of MM in RD cells (**E**) and Vero cells (**F**) were determined. All data are presented as mean ± SEM from three independent experiments. Statistical significance was evaluated using one-way ANOVA. *P* values are indicated as follows: *P < 0.05, ***P < 0.001, ****P < 0.0001; ns, not significant.

To identify the compounds from *Smilax china* with anti-EV71 activity, we screened the inhibitory effects of 31 extracts with EV71-GFP infection. RD cells were infected with EV71-GFP for 2 h and then treated with various compounds or the positive control ribavirin. Cells were collected at 24 hours post-infection (hpi), and the percentage of GFP-positive cells was determined by flow cytometry to calculate the inhibition rate relative to vehicle control cells. Four compounds exhibited inhibitory effects exceeding 30% to EV71-GFP infection. Among them, only SC-11 showed the most potent inhibition, with a 71.8% inhibition rate, surpassing the positive control ribavirin (53%) (Fig. 1A). MM is identified to be a phenolic compound, and the chemical structure of it is shown in Fig. 1B.

Prior to investigating the detailed inhibitory effects, we evaluated the cytotoxicity of MM in target cells. Vero and RD cells were treated with various concentrations of MM for 24 h, and the cell viability was assessed using the CCK-8 assay. The results showed that MM exhibited no cytotoxicity at concentrations below 50 μM in both cell lines (Fig. 1C and E). The CC₅₀ values of MM were determined to be 176.8 μM in RD cells (Fig. 1D) and 189.8 μM in Vero cells (Fig. 1F). The favorable safety profile suggests that MM has potential for development as an EV71 inhibitor.

### MM significantly suppresses EV71 infection

**Fig 2:**
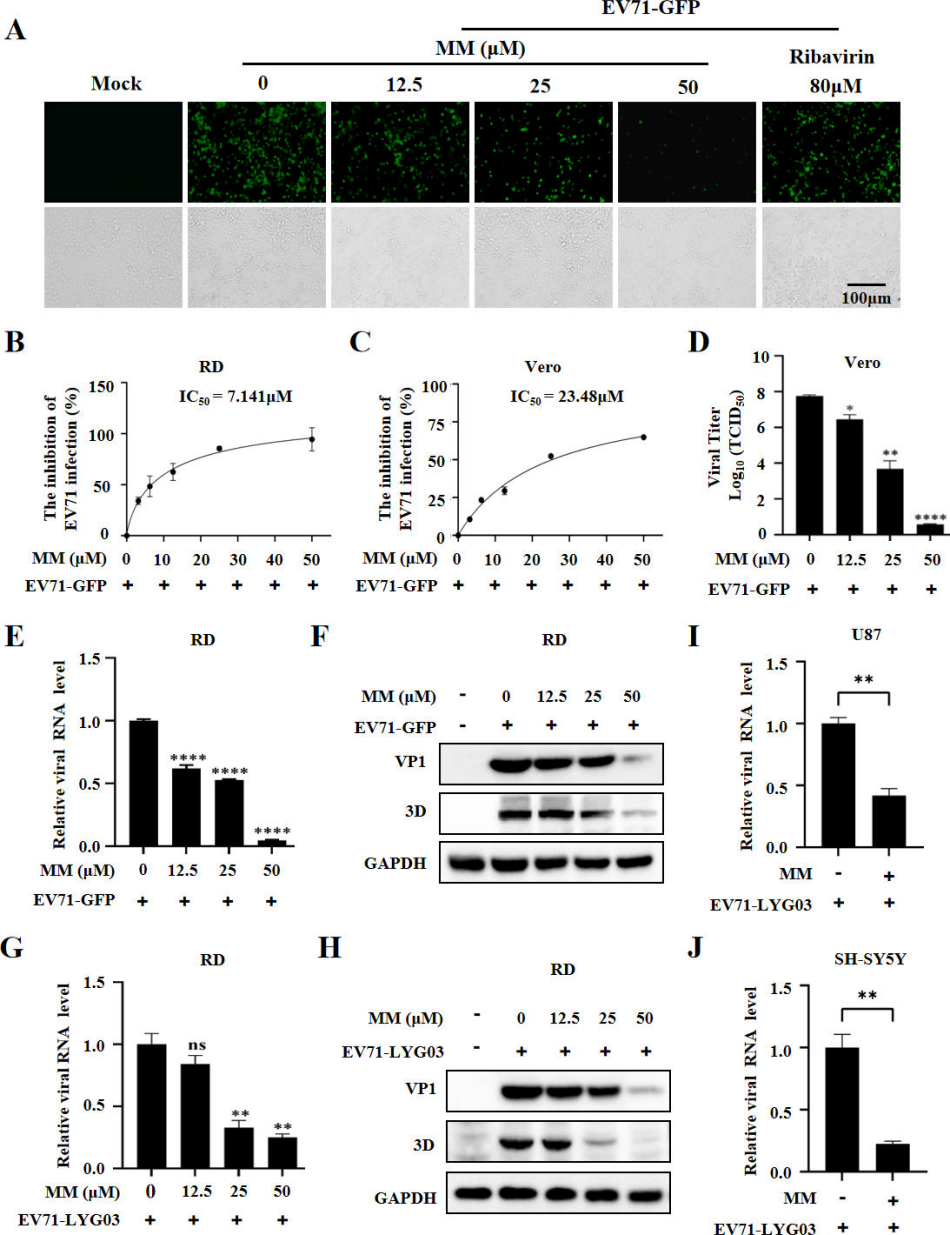
The effect of MM on EV71 infection. RD cells were infected with EV71-GFP (MOI = 1) for 2 h and subsequently treated with MM at concentrations of 0, 12.5, 25, 50 μM, or with 80 μM ribavirin. At 24 hpi, cytopathic effects and GFP-positive cells were observed under a microscope (**A**). The percentage of GFP-positive cells was quantified by flow cytometry, and the inhibition rate of EV71 infection (%) was calculated as [1 – FITC (treatment)/FITC (vehicle control)] × 100. IC_50_ values were determined based on the inhibition rates (**B**). Similar experiments were performed with Vero cells, and the IC_50_ of MM was measured and calculated (**C**). In parallel experiments, RD cells infected with EV71-GFP (MOI = 1) were treated with MM at concentrations of 0, 12.5, 25, and 50 μM. At 24 hpi, the supernatants were collected to determine TCID_50_ (**D**). EV71-GFP (MOI = 1) or EV71-LYG03 (MOI = 0.5) infected cells were treated with MM at concentrations of 0, 12.5, 25, and 50 μM. At 24 hpi, cells were harvested for the detection of EV71 RNA by RT-qPCR (**E, G**) and VP1 or 3D protein expression by Western blot (**F, H**), with GAPDH serving as an internal loading control. Magnification: 100× (scale bar: 100 μm). Both U87 (**I**) and SH-SY5Y (**J**) cells were infected with EV71-LYG03 (MOI = 1), followed by treatment with 50 μM MM or 0.1% DMSO as a vehicle control. At 24 hpi, viral RNA levels were determined by RT-qPCR. All data are presented as mean ± SEM of triplicate measurements. Statistical significance was evaluated using one-way ANOVA. *P* values are indicated as follows: *P < 0.05, **P < 0.01, ****P < 0.0001; ns, not significant.

We further validated the effect of compound MM on EV71 infection. RD and Vero cells were infected with EV71-GFP for 2 h and subsequently treated with a series of concentrations of MM. At 24 hpi, the infection rates were monitored and quantified by flow cytometry. The results showed that the proportion of GFP-positive cells decreased in an MM dose-dependent manner (Fig. 2A). Based on the flow cytometry data, the inhibition rates were calculated to determine the IC_50_ values. The results showed that the IC_50_ of MM is 7.141 μM in RD cells (Fig. 2B) and 23.48 μM in Vero cells (Fig. 2C), which is significantly lower than the CC_50_ of it in two cell lines.

Since the virus production is a key indicator of infection efficiency, we measured the EV71 titer in the supernatant of RD cells treated with a series of concentrations of MM following EV71-GFP infection. The results showed that viral titers were reduced by MM treatment in a dose-dependent manner (Fig. 2D). We also assessed the levels of viral RNA and viral protein VP1 or 3D in the lysates of RD and Vero cells under similar treatment. The results showed that the levels of viral RNA (Fig. 2E) and viral proteins (Fig. 2F) were decreased in an MM dose-dependent manner. Furthermore, the wild-type strain EV71 (EV71-LYG03) infection experiment confirmed these findings (Fig. 2G and H).

Given the neurotropism of EV71, which mainly invades the central nervous system and causes severe neurological complications (28), human glioma cell line U87 and neuroblastoma cell line SH-SY5Y were used to detect the anti-EV71 capability of MM. U87 cells and SH-SY5Y cells were infected with wild type EV71-LYG03, followed by treatment with 50 μM MM or 0.1% DMSO as a vehicle control, and the results showed that the level of viral RNA also decreased in MM treated brain cells (Fig. 2I and J). Collectively, these results demonstrate that MM significantly suppresses EV71 infection.

### MM mainly inhibits the attachment of EV71

**Fig 3:**
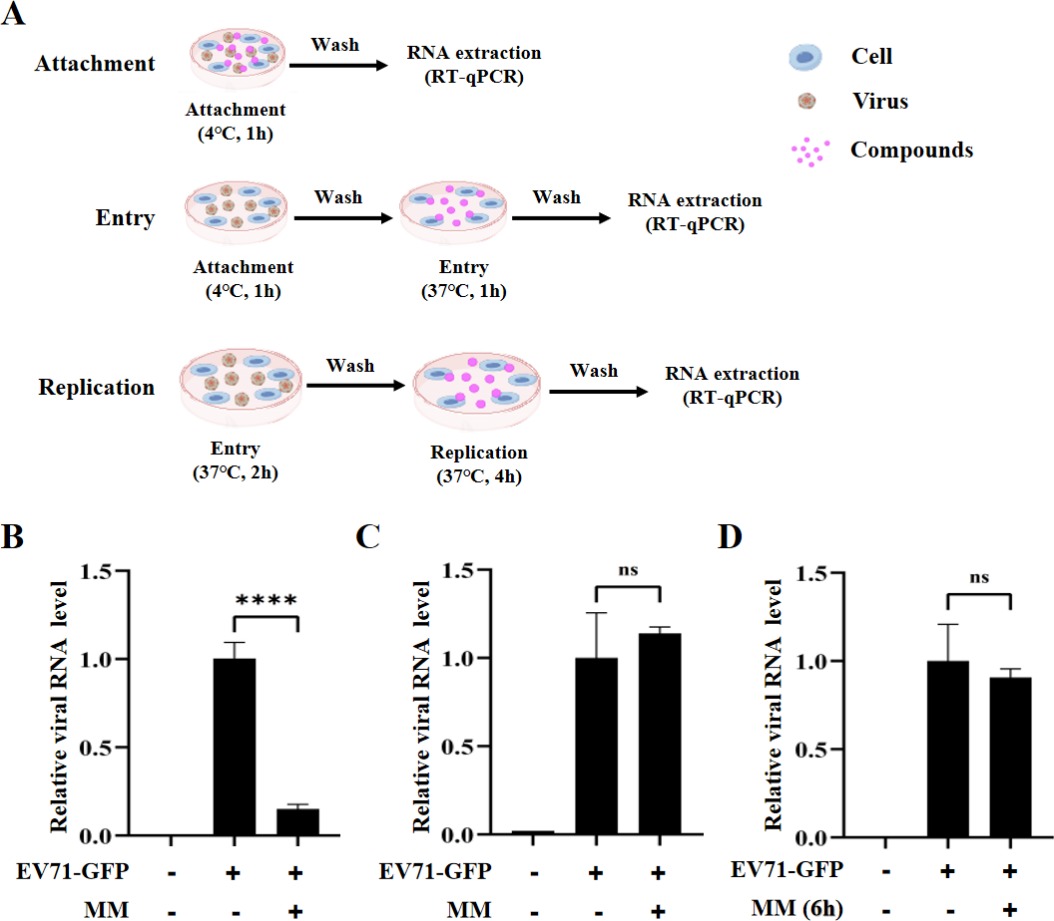
The influence of MM on the viral replication stage. (**A**) The diagram of the attachment, entry, and replication stages detection assay. (**B**) For the attachment assay, the pre-cooled RD cells were incubated with the mixture of EV71-GFP (MOI = 5) and 50 μM MM at 4°C for 1 h. After being washed with cold PBS, the viral RNA bound to the virus was determined by RT-qPCR. (**C**) For the entry assay, RD cells were incubated with EV71-GFP (MOI = 5) at 4°C for 1 h, were washed with PBS, and treated with 50 μM MM at 37°C for 1 h. The viral RNA was determined by RT-qPCR. (**D**) For the replication assay, RD cells were infected with EV71-GFP (MOI = 1) at 37°C for 2 h, and then the cells were washed and treated with 50 μM MM for 4 h. The cells were collected, and the viral RNA was detected by RT-qPCR. All data are presented as mean ± SEM of triplicate measurements. Statistical significance was evaluated using one-way ANOVA. *P* values are indicated as follows: **P < 0.01, ***P < 0.001, ****P < 0.0001; ns, not significant.

To determine which stage of EV71 life cycle is affected by MM, a time-of-addition assay was performed. As shown in Fig. 3A, MM was added to RD cells at various time points before, during, and after EV71-GFP infection. For attachment inhibition analysis, EV71-GFP and MM were pre-mixed and then incubated with cooled RD cells at 4°C for 1 h. The results showed that the viral RNA levels in the cells treated with MM were significantly lower compared to the vehicle group (Fig. 3B). For entry inhibition analysis, RD cells were first incubated with EV71-GFP at 4°C for 1 h, followed by treatment with MM at 37°C for 1 h to permit synchronous viral entry. Viral RNA quantification revealed that MM did not affect the entry of EV71 into host cells (Fig. 3C). For replication inhibition analysis, the virus might finish one replication cycle within 6 h (29). RD cells were infected with virus at 37°C for 2 h and then treated with MM for 4 h. Viral RNA quantification showed that MM treated cells contained similar levels of viral RNA to the vehicle treated cells, suggesting that MM does not interfere with the replication stage of EV71 (Fig. 3D). Collectively, these results suggest that MM primarily suppresses the attachment of EV71 to host cells.

### MM inhibits the attachment of EV71 by targeting virus

**Fig 4:**
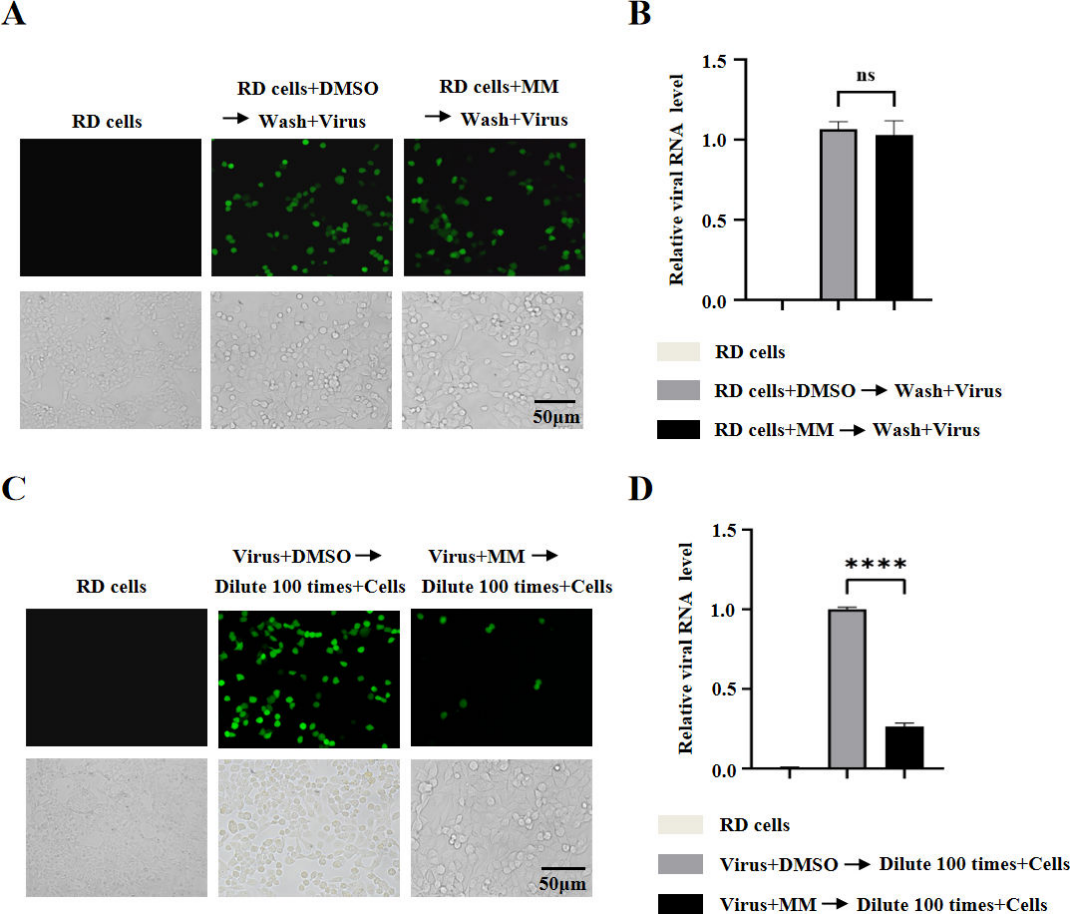
The effect of MM on virus or cells. RD cells were treated with 50 μM MM at 37°C for 1 h; after washing, they were infected with EV71-GFP (MOI = 1) for 24 h at 37°C. The proportion of GFP-positive cells and CPE was observed under a fluorescence microscope and bright field microscope, respectively (**A**), and viral RNA was quantified by RT-qPCR (**B**). EV71-GFP (MOI = 1) was incubated with 50 μM MM at 4°C for 4 h, was diluted 100-fold to minimize residual compound effects, and subsequently used to infect RD cells for 24 h. The proportion of GFP-positive cells and CPE was observed (**C**), and viral RNA (**D**) was detected. All data of viral RNA are presented as mean ± SEM of triplicate measurements. Magnification: 200× (scale bar: 50 μm). Statistical significance was evaluated using one-way ANOVA. *P* values are indicated as follows: ****P < 0.0001; ns, not significant.

To determine whether the target of MM is the host cell or the virion, a time-of-removal assay was performed. First, RD cells were pre-treated with MM or vehicle at 37°C for 1 h, followed by the removal of the compound and infection with EV71-GFP for 24 h. The GFP-positive cells (Fig. 4A) and viral RNA (Fig. 4B) detection results showed no significant reduction in MM treated cells compared to the vehicle control, indicating that pretreatment of cells with MM does not affect EV71 infection. Next, EV71-GFP was incubated with MM at 4°C for 4 h. Subsequently, the mixture was diluted 100-fold to minimize the potential effect of residual MM on cells, and then they were added to RD cells for 24 h infection. Flow cytometry and RT-qPCR analysis results showed a significant decrease in the infected cells ratio and viral RNA level in the cells infected with MM treated virus compared with the vehicle control (Fig. 4C and D). These results indicate that MM inhibits viral attachment by directly targeting EV71 rather than host cell.

### MM also suppresses the attachment of CA16

**Fig 5:**
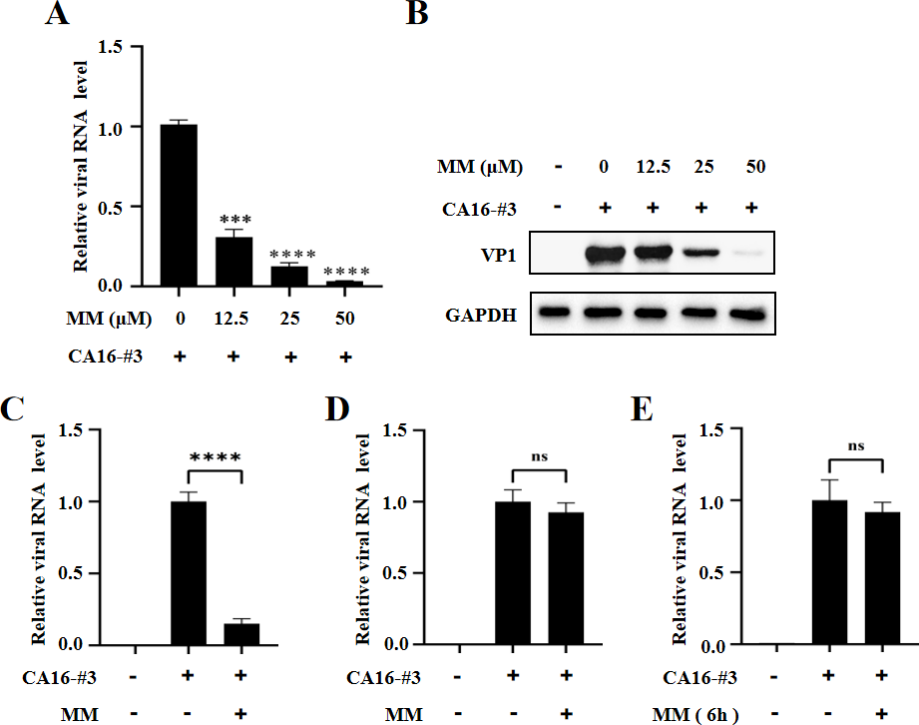
The influence of MM on CA16 infection. (**A**) Vero cells were infected with CA16-#3 (MOI = 1) for 2 h, washed with PBS, and treated with different concentrations of MM. After 24 h of infection, the viral RNA was detected by RT-qPCR. (**B**) The expression of Vp1 protein was detected by Western blot. (**C**) The attachment phase was performed as in Fig. 3B. (**D**) The entry phase was performed as in Fig. 3C. (**E**) The replication phase was performed as in Fig. 3D. All data are presented as mean ± SEM of triplicate measurements. Statistical significance was evaluated using one-way ANOVA. *P* values are indicated as follows: **P < 0.01, ***P < 0.001, ****P < 0.0001; ns, not significant.

Given the structural and morphological similarities among enteroviruses and the fact that CA16 is one of the major causative agents of HFMD, we further investigated the antiviral activity of MM to CA16. Similar to the experiments with EV71, Vero cells were infected with CA16-#3 for 2 h and then treated with various concentrations of MM. At 24 hpi, the levels of viral RNA and VP1 protein were detected, and the results showed that MM also inhibited CA16-#3 infection in a dose-dependent manner (Fig. 5A and B). A time-of-addition assay as employed for EV71 was also performed for CA16 infection, and the levels of viral RNA detection showed that MM also primarily inhibited CA16 attachment to host cells (Fig. 5C) and had no effect on viral entry (Fig. 5D) and the replication stage (Fig. 5E). These findings indicate that MM also suppresses CA16 infection predominantly by interfering with the viral attachment.

### MM showed favorable safety in neonatal mice

**Fig 6:**
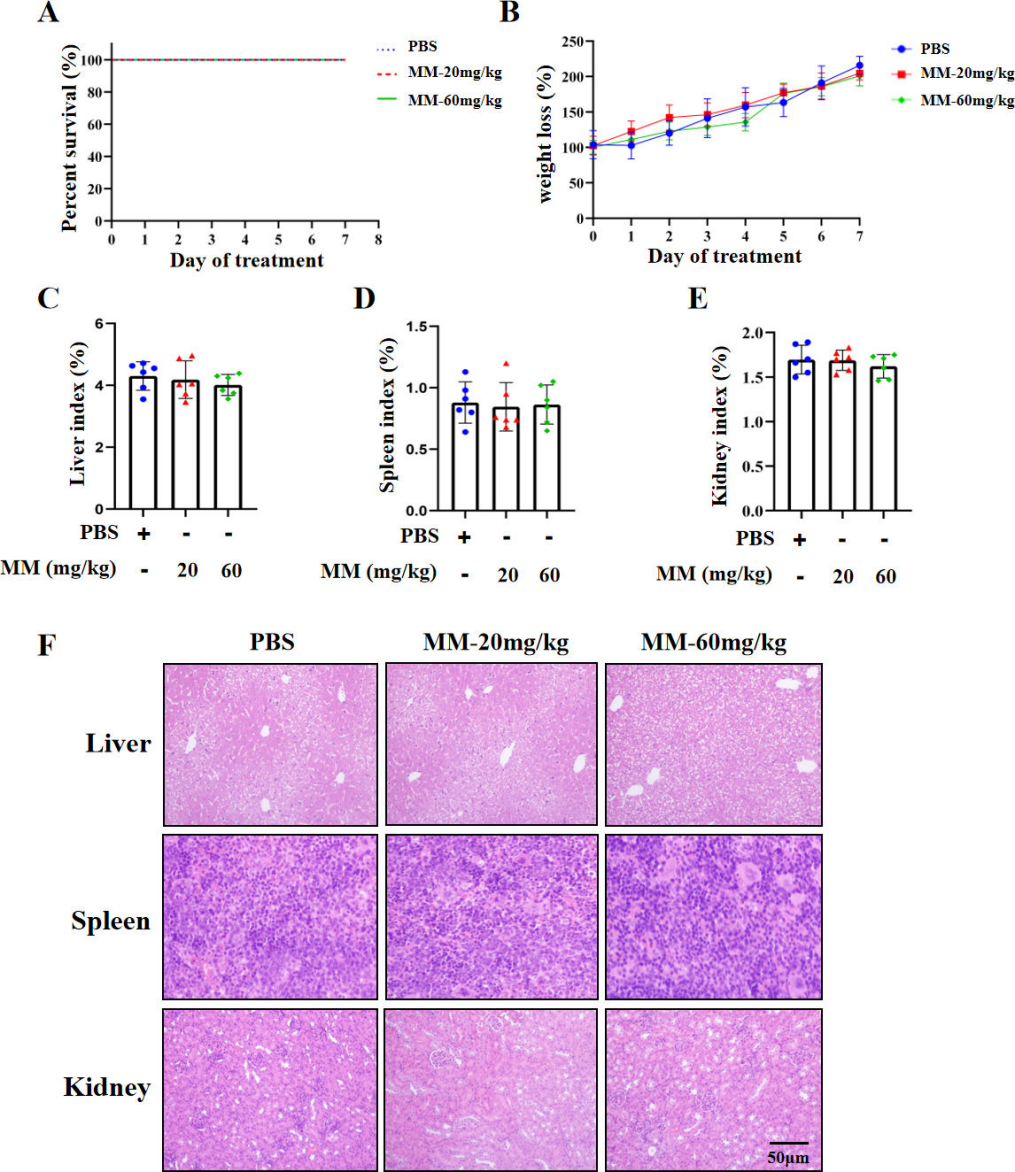
The evaluation of the acute toxicity of MM in newborn ICR mice. Three-day-old suckling mice were divided into three groups (6 mice per group): the PBS control group, the MM-treated group receiving 20 mg/kg or 60 mg/kg. The survival rate (**A**) and body weight changes (**B**) were monitored and recorded. Liver index (**C**), spleen index (**D**), and kidney index (**E**) were calculated as the ratio of individual organ weight to total body weight. Histopathological changes in the liver, spleen, and kidney were assessed by H&E staining (**F**). Magnification: 200× (scale bar: 50 μm).

To evaluate the therapeutic toxicity of MM, neonatal mice were administered intraperitoneal injections of MM at 20 mg/kg or a triple dose of 60 mg/kg daily for 7 consecutive days. MM treated mice showed 100% survival rate (Fig. 6A) and no significant effect on body weight following either dose regimen (Fig. 6B). The organ coefficients of liver, spleen, and kidney tissues were determined, and the results showed no significant differences among two doses of MM treated and the control mice (Fig. 6C and E). Additionally, histopathological examination by H&E staining revealed no tissue damage in MM treated mice compared with control (Fig. 6F). These results indicate that MM exhibits favorable safety profiles in newborn mice.

### MM significantly inhibits EV71 infection *in vivo*

**Fig 7:**
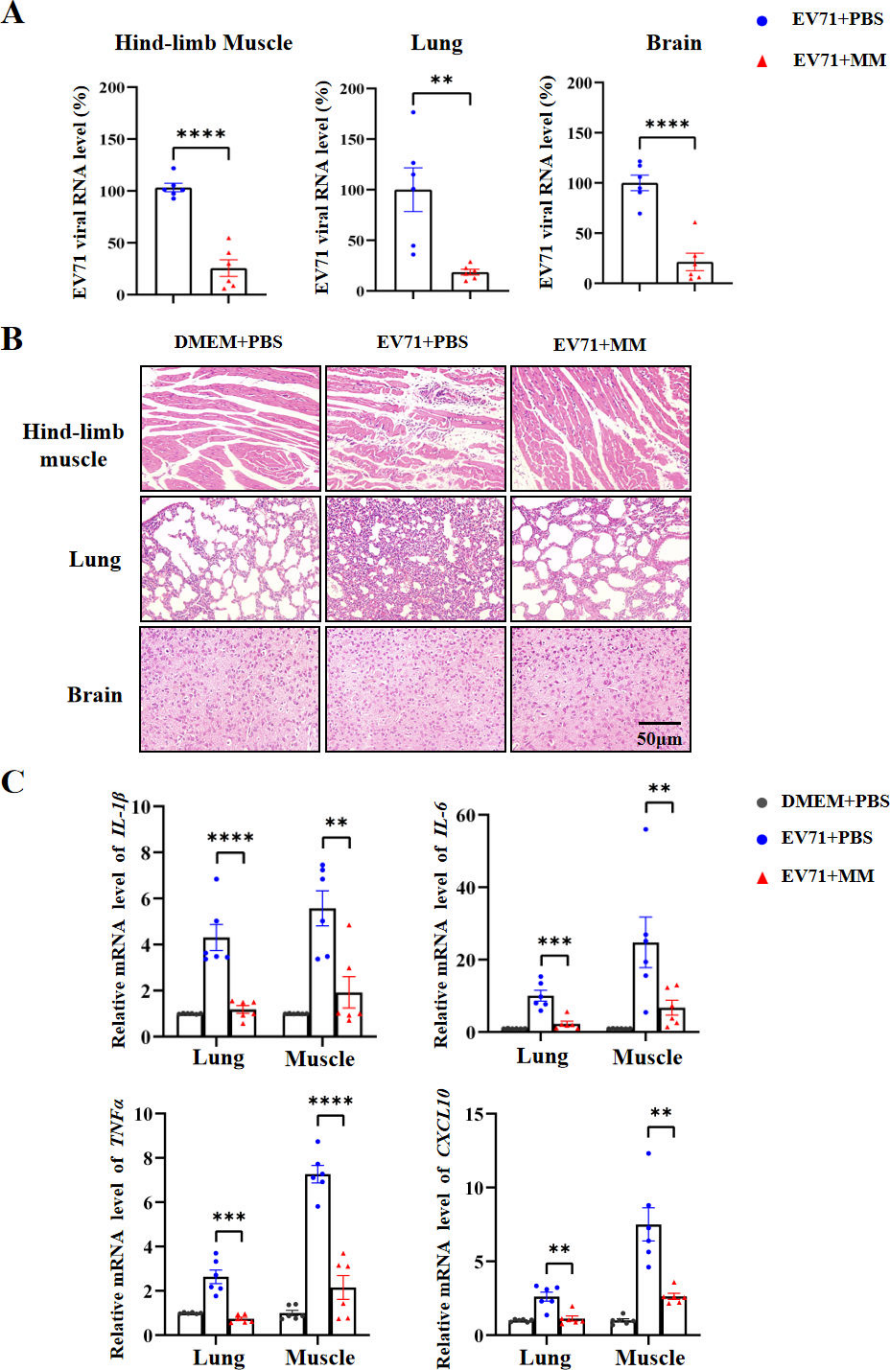
The inhibitory effect of MM on EV71 infection in newborn ICR mice. Three-day-old mice were divided into three groups (*n* = 6). One group served as the negative control, which was treated with DMEM and PBS (DMEM + PBS). The other two groups were challenged with 10 μL of virus solution containing 10⁷ TCID₅₀ EV71-LYG03; subsequently, one group was administered MM at 20 mg/kg once daily for 5 consecutive days and designated as EV71 + MM group, while the other group received PBS and served as and designated as EV71 + PBS group. On day 6 post-infection, hindlimb muscles, lungs, and brain were collected from mice in all three groups. Viral RNA levels in hindlimb muscles, lungs, and brain (**A**) were quantified by RT-qPCR. Histopathological changes in hindlimb muscles, lungs, and brain were evaluated by H&E staining (**B**). The relative mRNA levels of *IL-1β*, *IL-6*, *TNFα, and CXCL10* in these tissues were determined by RT-qPCR (**C**). Magnification: 200× (scale bar: 50 μm). All data are presented as mean ± SEM of triplicate measurements. Statistical significance was evaluated using one-way ANOVA and *t*-test. **P < 0.01, ***P < 0.001, ****P < 0.0001.

We further used newborn ICR mice to confirm the inhibitory effect of MM on EV71 infection. Three-day-old ICR mice were randomly divided into three groups (*n* = 6); 6 mice and 12 mice were intraperitoneally injected with PBS (as control) or EV71-LYG03, respectively; the infected mice were randomly divided into two groups and treated with MM or DMEM for 5 days, and then the mice were sacrificed at 6 dpi. The viral RNA levels in the hindlimb muscles, lungs, and brain were detected with RT-qPCR, and the results showed that MM significantly reduced viral RNA levels in these tissues (Fig. 7A). Histopathological examination of hindlimb muscles, lungs, and brain by H&E staining. Compared with the uninfected mice, the lung tissues of the EV71-infected group showed extensive coagulative necrosis, fusion of alveoli forming pulmonary bullae, and a large number of inflammatory cell infiltrations. At the same time, the hind limb muscle tissues showed obvious muscle fiber dissolution and tissue damage. In the brain tissue, some neurons undergo nuclear shrinkage and pale staining, and the cell morphology becomes wrinkled. And MM treatment significantly alleviated the lung lesions, hind limb muscle, and brain damage caused by EV71 infection (Fig. 7B). Furthermore, we assessed the expression profiles of key pro-inflammatory cytokines and antiviral immune mediators. We found that MM treatment significantly attenuated the mRNA levels of *IL-1β*, *IL-6*, *TNFα*, and *CXCL10* induced by EV71 infection (Fig. 7C) in both hindlimb muscle and lung tissues. These results demonstrate that MM effectively suppresses EV71 infection and mitigates virus-induced tissue damage in newborn mice.

## DISCUSSION

EV71 and CA16 are the main pathogens causing HFMD (30); to find the inhibitor for these viruses will provide the specific and effective drugs for HFMD treatment. In this study, we identified and demonstrated that MM effectively inhibits EV71 and CA16 infection and revealed that MM primarily suppresses viral attachment to host cells by targeting the virion. Furthermore, MM exhibited favorable safety profiles and significant antiviral efficacy in newborn mice infected with EV71.

MM is a phenolic compound primarily found in the bark of Moraceae plants (31). Numerous studies have reported that phenolic compounds possess potential antiviral properties. For instance, tea polyphenols effectively inhibit PRRSV infection by targeting multiple stages of the viral life cycle, including attachment, internalization, replication, and release (32). Resveratrol exerts its antiviral activity by interfering with the assembly of influenza virus (33). Our group has previously demonstrated that resveratrol oligomers can inhibit SARS-CoV-2 infection by suppressing the activity of cathepsin L, thereby blocking viral entry (34). Additionally, curcumin has been shown to inhibit the infection of influenza virus, ZIKV, and hepatitis C virus (HCV) (35–37). In this study, we identified and demonstrated that MM can inhibit the infection of EV71 and CA16 mainly by interfering with the attachment of virus. Although individual phenolic compound exerts antiviral effect via distinct mechanism, collectively these findings suggest that phenolic compounds represent promising candidates for the treatment of viral infectious diseases, and they can be developed into broad-spectrum antiviral agents.

MM has been reported containing antiviral activity on a variety of viruses although the underlying mechanisms remain to be elucidated. Studies have demonstrated that MM is able to suppress HCV replication by inhibiting the activity of NS3 helicase (22). MM also exhibits antiviral effects on Herpes Simplex Virus (38) and Human Immunodeficiency Virus Type 1 (39), but the underlying mechanisms remain unclear. In this study, we first found that MM is able to inhibit the infection of EV71 and CA16 and revealed that MM primarily inhibits the attachment stage of EV71 and CA16. It is reported that some phenolic compounds suppress virus infection by inhibiting the binding of virus to receptors, such as epigallocatechin gallate prevents influenza virus attachment to sialic acid receptors on the host cell surface by binding to the hemagglutinin protein of the virus (40). Additionally, tannic acid was also reported to prevent viral attachment to host cells by directly binding to the capsid proteins of norovirus (41). Although our study confirmed that MM inhibits the attachment of EV71 and CA16 by targeting the virus itself rather than host cells, its specific mechanism of action remains unclear. We speculate that this inhibitory effect may involve multiple pathways; as a lipophilic small-molecule compound, MM can directly bind to viral surface proteins through steric hindrance to block virus-receptor interactions, alter cell membrane fluidity and lipid raft structure to reduce the accessibility of viral receptors, or disrupt the integrity of viral particles to reduce the number of infectious virus available for attachment. The exact molecular mechanism requires further in-depth investigation.

A large number of medicinal phenolic compounds originate from plants, and most of them can also be synthesized. Their core structure consists of a benzene ring attached to one or more hydroxyl groups (-OH) (42). Although some of phenolic compounds are highly toxic, whereas many of them are relatively safe and may even confer health benefits in humans. As previously mentioned, tea polyphenols are an example of such beneficial compounds (43); resveratrol and curcumin exhibit significant benefits for human health maintenance (44). We systematically evaluated the safety of MM at both cellular and animal levels. The results showed that the concentrations below 50 μM of MM exhibit no cytotoxicity in two target cells. The doses of MM used in aged mice for the inflammation inhibition study were 20 mg/kg and 60 mg/kg, respectively, and showed good drug safety (20). In this study, we administered the same dose of MM to 3-day-old mice for 7 consecutive days, and the results showed that both doses of MM showed well safety in neonatal mice. The favorable safety profile suggests that MM has the potential to be developed into a clinical therapeutic agent.

Although the dose of MM *in vivo* seems to be higher than in cell experiments, such a difference is quite common in preclinical antiviral studies and can be explained by key pharmacokinetic factors. *In vitro*, MM acts directly on virus particles and cells in a controlled environment, unaffected by physiological processes such as absorption, distribution, metabolism, and excretion (ADME) (45). In contrast, *in vivo*, there might be only a small portion of the administered dose may reach the systemic circulation and target tissues. Previous pharmacokinetic studies of MM indicate that its half-life is short and its oral bioavailability is moderate (46). Additionally, MM has a clear safety with high doses in our mice experiment, which allows for the use of higher doses *in vivo* to overcome pharmacokinetic limitations, ensuring effective antiviral activity while maintaining a good safety range.

We further investigated the antiviral function of MM in newborn mice at 20 mg/kg, and the viral RNA detection showed that MM significantly reduced the viral load in the lungs and muscles of EV71-infected mice. The time-of-addition assays in cells showed that MM mainly suppresses the attachment of EV71 and CA16 and also suppresses the replication of the virus. Combined with the significant inhibitory effect *in vivo*, we guess that MM might target multiple points to suppress EV71 infection. The replication process is highly complex, involving viral nucleic acid replication and protein synthesis and interaction between virus and host (47). Regarding the possible inhibitory effect of MM on the viral replication stage, we propose two potential mechanisms. First, MM may directly inhibit the activity of key viral proteases involved in replication. Second, it may activate the antiviral signaling pathways of cells to restrict virus infection and replication (48). Further investigation is needed to validate these hypotheses and clarify the inhibitory effects of MM on EV71 infection.

We also observed in the mouse experiment that tissue damage induced by EV71 infection was almost completely repaired, and the levels of inflammatory cell infiltration and cytokines IL-1β and IL-6 were markedly reduced in MM-treated mice. It has been reported that MM can suppress the inflammatory response by inhibiting the NF-κB signaling pathway (20, 46, 49), and viral RNA can also activate the NF-κB signaling pathway by binding to TLRs. Excessive activation of the inflammatory response can cause tissue damage and lead to the development of various inflammatory diseases (50). These results suggest that MM not only inhibits viral infection but also suppresses the inflammatory response triggered by viral infection, further supporting its strong pharmaceutical potential for HFMD treatment.

### Conclusions

This study demonstrated that MM can significantly inhibit EV71 and CA16 infection by suppressing viral attachment to the target cells. Furthermore, EV71 infection in neonatal a mouse model revealed that MM contains favorable safety and significantly inhibits virus infection and might have the ability to suppress the inflammatory response to promote tissue damage repair. These findings suggest that MM has strong potential for further development as an antiviral agent for the treatment of HFMD induced by EV71 and CA16 infection.

## Data Availability

Data will be made available on request.
